# Characterization of the RNA-binding properties of the triple-gene-block protein 2 of *Bamboo mosaic virus*

**DOI:** 10.1186/1743-422X-6-50

**Published:** 2009-05-07

**Authors:** Hsiu-Ting Hsu, Yang-Hao Tseng, Yuan-Lin Chou, Shiaw-Hwa Su, Yau-Heiu Hsu, Ban-Yang Chang

**Affiliations:** 1 Institute of Biochemistry, National Chung-Hsing University, Taichung 40227, Taiwan, PR China; 2 Department of Soil and Environmental Science, National Chung-Hsing University, Taichung 40227, Taiwan, PR China; 3 Graduate Institute of Biotechnology, National Chung-Hsing University, Taichung 40227, Taiwan, PR China

## Abstract

The triple-gene-block protein 2 (TGBp2) of *Bamboo mosaic virus *(BaMV) is a transmembrane protein which was proposed to be involved in viral RNA binding during virus transport. Here, we report on the RNA-binding properties of TGBp2. Using tyrosine fluorescence spectroscopy and UV-crosslinking assays, the TGBp2 solubilized with Triton X-100 was found to interact with viral RNA in a non-specific manner. These results raise the possibility that TGBp2 facilitates intracellular delivery of viral RNA through non-specific protein-RNA interaction.

## Findings

*Bamboo mosaic virus *(BaMV) is a single-stranded, positive-sense RNA virus. Its genomic RNA has three partially overlapping open reading frames, called triple gene block (TGB), located between the coding sequences for the replicase and capsid protein [1]. The TGB-encoded proteins are referred to as TGBp1, TGBp2 and TGBp3 according to their positions [2] and are required for virus movement in the host plant [3-6]. The TGB proteins are found in several different viral genera. On the basis of amino acid sequence comparisons of the TGB proteins, the TGB-containing viruses have been classified into hordei-like and potex-like viruses [7]. *Bamboo mosaic virus *is a potex-like virus.

The functions of each TGB protein have been investigated. TGBp2 is an integral membrane protein with two transmembrane helices [8] and a topology with both its N- and C-terminal tails exposed to the outer surface of endoplasmic reticulum (ER) and the central loop in the lumen of ER [9,10]. Inhibition of virus movement by mutations disrupting the transmembrane helices of *Potato virus X *(PVX) TGBp2 indicated that ER association is important for the functioning of TGBp2 (8). Moreover, the PVX TGBp2 is able to induce the formation of granular vesicles derived from the ER, which align on actin filaments [11]. Mutations in the central loop region of PVX TGBp2 eliminate the formation of granular vesicles and inhibit the cell-to-cell movement of virus [12]. In addition, the PVX TGBp2 is able to increase the size exclusion limit of plasmodesmata (PD) [13], probably through its association with host interacting proteins (TIPs) which in accompany with β-1, 3-glucanase regulate callose degradation [14].

The membrane-associated TGBp2 is thought to assist the intracellular transport of the viral ribonucleoprotein (RNP) complex to the PD by a subcellular translocation process via cytoskeleton and is assumed to function through protein-protein or protein-RNA interactions [15,16]. The RNA-binding activity of a thioredoxin-fused *Potato mop-top virus *(PMTV) TGBp2 has been detected using Northwestern blot [15]. However, RNA binding of TGBp2 in aqueous solution has not been studied. To confirm that TGBp2 is able to bind viral RNA and to gain insight into the RNA-binding properties of TGBp2, we prepared unfused TGBp2 [9] and His_6_-tagged TGBp2 of BaMV to characterize their RNA-binding properties using tyrosine fluorescence spectroscopy and zero-length UV-crosslinking assay.

**Figure 1:**
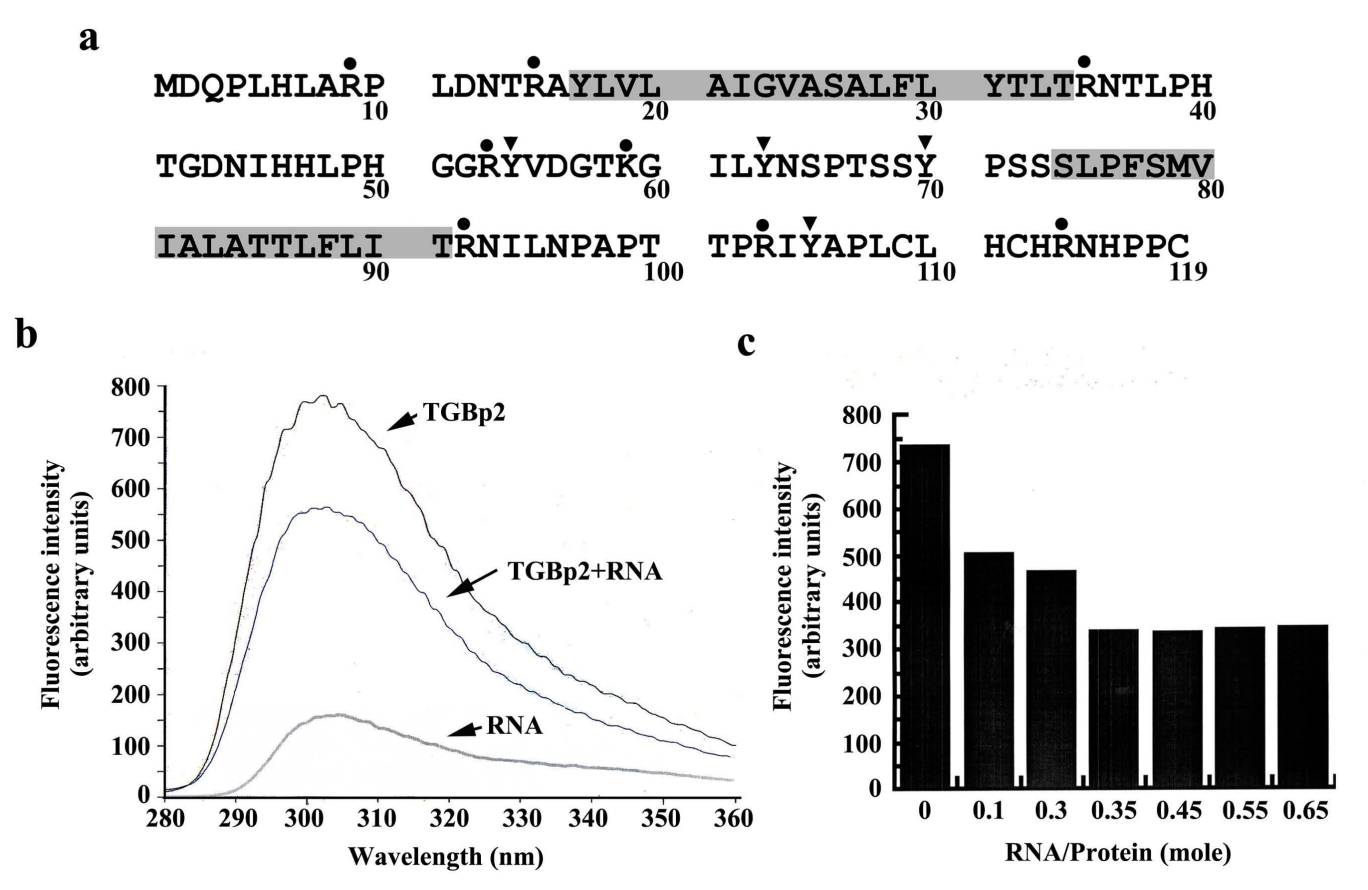
**Spectroscopic analyses of the interaction between the unfused TGBp2 and viral RNA**. A. The amino acid sequence of TGBp2. The TGBp2 protein contains an N-terminal tail, a central loop in between the two transmembrane helices and a C-terminal tail as predicted by ExPASy proteomic tools (HMMTOP 2.0). The two transmembrane helices are highlighted by gray box. (•), the positions of basic amino acid residues replaced with Ala. (▼), the positions of Tyr-to-Ala substitutions. B. Effect of viral RNA on the intrinsic tyrosine fluorescence of Triton X-100-solubilized TGBp2. The Triton X-100-solubilized TGBp2 (2 μM or 25.3 μg/ml) were excited with UV in the presence (at a molar ratio of viral RNA to TGBp2 of 0.35:1) or absence of viral RNA before measurement of tyrosine fluorescence. C. Effect of viral RNA concentration on the intrinsic tyrosine fluorescence of Triton X-100-solubilized TGBp2. Samples of viral RNA and TGBp2 were mixed in various molar ratios, excited with UV and measured for tyrosine fluorescence. In both (B) and (C), the tyrosine fluorescence of TGBp2 was measured at 303 nm after excitation with UV at a wavelength of 280 nm.

In order to test whether the BaMV TGBp2 is able to bind viral RNA, intrinsic fluorescence measurement was conducted. This method has been used to identify amino acid residues essential for RNA binding of influenza virus nucleoprotein [17]. In this analysis, the unfused TGBp2 was solubilized with Triton X-100, a mild non-ionic detergent, as previously described [9]. The solubilization allows the membrane protein to adopt a topology mimicing that of the same protein residing in lipid bilayers [18,19]. In other words, the two transmembrane helices of TGBp2 are supposed to be bound by Triton X-100. And the two tyrosine residues in the central loop and the one in the C-terminal tail domain are exposed (Figure 1A). Then, the viral RNA fragment (220 bases in length) derived from the 3' end of BaMV genome was synthesized using *in vitro *transcription and the linearized pBaMV plasmid as a template [20]. After mixing the Triton X-100-solubilized TGBp2 for 5 min with the viral RNA fragment and excitation of the sample with UV at a wavelength of 280 nm, tyrosine fluorescence was measured at 303 nm using an F-4500 FL Spectrophotometer. We expected to see a reduction in tyrosine fluorescence if TGBp2 is able to come closer to viral RNA. As expected, we observed a 26% reduction in maximal tyrosine fluorescence of TGBp2 after incubation with the viral RNA fragment at a molar ratio of 1:3 (RNA:TGBp2) (Figure 1B). These results suggested that TGBp2 is in close proximity to the RNA, resulting in quenching of the tyrosine fluorescence. We then studied the effect of changing the molar ratio of the viral RNA fragment to TGBp2 on the tyrosine fluorescence quenching. Decrease in tyrosine fluorescence was observed as the molar ratio of viral RNA to TGBp2 was increased from 0:1 to 0.35:1; thereafter the fluorescence became relatively constant (Figure 1C), suggesting that TGBp2 is able to complex with the tested viral RNA in a 3:1 stoichiometry.

**Figure 2:**
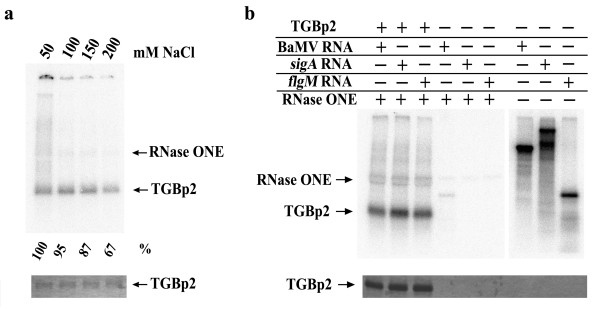
**The RNA-binding properties of unfused TGBp2**. A. Effect of salt concentration on the RNA-binding activity of unfused TGBp2. After UV-crosslinking with TGBp2, the ^32^P-labeled viral RNA was digested with RNase ONE. The protein sample was then run on a Tricine SDS-polyacrylamide gel before autoradiography. B. Non-specific RNA binding of TGBp2. The BaMV RNA (220 nucleotides), *sigA *RNA (400 nucleotides), and *flgM *RNA (164 nucleotides) were synthesized using the linearized pBaHB, p*sigA*-100-2.4 and pEd21d-*flgM *plasmids as templates, respectively. The RNA-binding assay was carried out using the same method as described in A. Both autoradiography (upper panel) and Coomassie blue staining (bottom panel) of TGBp2 are shown for each panel.

To confirm that TGBp2 interacts with the viral RNA fragment, zero-length UV-crosslinking assay was performed under various NaCl concentrations as used for assaying the RNA-binding activity of TGBp1 [20]. In the assay, 2.5 μg of the unfused TGBp2 solubilized with Triton X-100 was mixed with 15 ng of ^32^P-labeled viral RNA fragment. The mixture was incubated on ice for 15 min and irradiated with a Stratalinker (Stratagene) for 8 min at a distance of 8 cm from the light source (0.78 J/cm^2^). After UV crosslinking, the RNA was digested with 60 units of RNase ONE (Promega) at 37°C for 3 hours. TGBp2 was precipitated with acetone and separated on Tricine SDS-polyacrylamide gel. After staining and drying of the gel, autoradiography was performed. As shown in Figure 2A, the binding of TGBp2 to viral RNA in 200 mM NaCl was decreased to about 67% of that obtained in 50 mM NaCl (Figure 2A). The slight effect of salt concentration on RNA binding of TGBp2 indicated that salt bridge may, to a certain extent, participate in viral RNA binding of TGBp2.

To determine whether the unfused TGBp2 binds viral RNA in a specific or non-specific manner, two non-viral RNAs (the mRNAs of *sigA *and *flgM *genes from *Bacillus subtilis*) were synthesized *in vitro *using the same method as described above. The ability of TGBp2 to bind the two bacterial mRNAs (Figure 2B) indicated that TGBp2 interacts with RNA in a non-specific manner.

**Figure 3:**
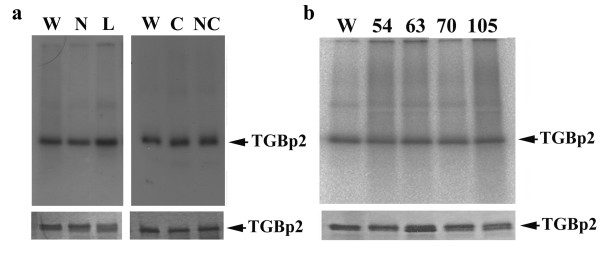
**Effect of amino acid substitutions on the RNA-binding activity of His_6_-TGBp2**. A, B. Effects of substitutions of basic amino acid residues and tyrosine residues, respectively, on the RNA-binding activity of TGBp2. Equal amount of wild-type or mutant TGBp2 was assayed for the RNA-binding activity using UV-crosslinking assay. The TGBp2 proteins were run on SDS-polyacrylamide gel. Autoradiography (upper panel) and Coomassie blue staining (bottom panel) of TGBp2 protein in the binding samples were performed. W, wild-type TGBp2. The mutant TGBp2 proteins were designated as follows: N (R-9 and R-15 mutated to A), L (R-45, R-53 and K-59 mutated to A), C (R-92, R-103 and R-114 mutated to A), NC (R-9, R-15, R-92, R-103 and R-114 mutated to A), 54 (Y-54 mutated to A), 63 (Y-63 mutated to A), 70 (Y-70 mutated to A), 105 (Y-105 mutated to A).

The slight effect of salt concentration on the RNA-binding activity of TGBp2 as presented in Figure 2A suggested that salt bridge between the positively charged amino acid residues of TGBp2 and the negatively charged phosphate backbone of viral RNA may, to a certain extent, be involved in the formation of TGBp2-viral RNA complex. To test this idea, basic amino acid residues, such as arginine (Arg) and lysine (Lys), in the N-terminal tail (residues 9 and 15), central loop (residues 45, 53, and 59) and C-terminal tail (residues 92, 103, and 114) domains of TGBp2 (Figure 1A) were mutated into alanine. Due to difficulties in expressing and purifying the mutant TGBp2, the wild-type and mutant TGBp2 were fused with 6 × His-tag. To construct the pJC2N plasmid used for the expression of wild-type His_6_-TGBp2, DNA fragment encoding the His_6_-TGBp2 was amplified by polymerase chain reaction using the pBL plasmid as a template [21] and the two primers, M2F and M2R (Table 1). The DNA fragment was then digested with *Hind*III and *Bam*HI and cloned into pT7-6 [22]. The His_6_-TGBp2 with Arg- or Lys-to-Ala substitutions was constructed using the QuikChange^® ^Site-Directed Mutagenesis Kit (Stratagene, La Jolla, California, USA) with pJC2N plasmid as template. The sequences of primers used for the mutagenesis are listed in Table 1. Methods used for expression of His_6_-TGBp2 and preparation of His_6_-TGBp2/Triton X-100 micelles were the same as those used for the unfused TGBp2. The effect of each mutation on non-specific RNA binding of His_6_-TGBp2 was analyzed by UV-crosslinking assay. As shown in Figure 3A, the RNA binding activity of His_6_-TGBp2 mutants having Arg- or Lys-to-Ala substitution(s) in the N-terminal tail, central loop or C-terminal tail domain was similar to that of the wild-type protein, suggesting that the basic amino acid residues of TGBp2 are not directly involved in non-specific RNA binding of TGBp2.

**Table 1:** Primers used for the construction of pJC2N and site-directed mutagenesis of His_6_-TGBp2

**Primer**	**Sequences of primers (from 5' to 3')**
HM2F	ATCAGAAAGCTTAAGAAGGAGATATACATATG*CACCACCACCACCACCAC*GACCAGCCTCTTCATCTG
M2R	CTCTTGGGATCCTCCTCAGTGTTTAGCATGGTG
R9A	CCTCTTCATCTGGCC**GCA**CCACCTGACAACACG
R15A	CCAGACCACCTGACAACACG**GCA**GCTTACTTAGTATTAGCTATAG
R35A	GTTCCTCTATACACTAACC**GCA**AATACCCTTCCACACACCGG
R53A	CCGCACGGGGGT**GCA**TACGTGGACGGCACC
R59A	GTACGTGGACGGCACC**GCA**GGAATTCTCTACAACAG
R92A	CCTTTTCCTCATCACC**GCA**AACATTCTCAACCCAGCC
R103A	CCCCCACCACACCT**GCA**ATCTATGCGCCCC
R114A	CCTCTGCTTGCATTGTCAC**GCA**AATCACCCACCATGCTAAAC
Y54A	CGCACGGGGGTAGG**GCA**GTGGACGGCACCAAAG
Y63A	GCACCAAAGGAATTCTC**GCA**AACAGCCCCACCTCCTC
Y70A	CAGCCCCACCTCCTCA**GCA**CCATCCTCATCTCTC
Y103A	CACCACACCTAGAATC**GCA**GCGCCCCTCTGCTTG

The underline nucleotide sequence indicates the *Hind*III or *Bam*HI restriction site. The italicised bases indicate the six His codons. The sites of Arg or Lys-to-Ala or Tyr-to-Ala substitution are shown in boldface.

It has been reported that aromatic amino acid residues can interact directly with single-stranded nucleic acids either by polar interactions or planar stacking with the exposed bases [17,23,24]. To test whether this is also true for tyrosine residues in TGBp2, we replaced the tyrosine residue(s) in the central loop (residues 54, 63, or 70) or C-terminal tail (residue 105) of His_6_-TGBp2 with alanine and analyzed the effects of these mutations on RNA binding of His_6_-TGBp2. No significant effect of tyrosine mutation on RNA binding of His_6_-TGBp2 was observed (Figure 3B), indicating that the tyrosine residues in both the central loop and C-terminal tail domains of TGBp2 are also not directly involved in non-specific RNA binding of TGBp2.

The lack of detectable effect of Arg- or Lys-to-Ala substitutions and Tyr-to-Ala substitutions on non-specific RNA binding of His_6_-TGBp2 (Figure 3) suggested that it is not specific amino acid residues but conformational property of TGBp2, which is responsible for the non-specific interaction between TGBp2 and viral RNA. On the basis of the known topological properties of TGBp2 [9], we propose that the self-assembly of TGBp2 through helical packing of transmembrane helices and/or disulfide linkages among the C-terminal tails of TGBp2 help to provide the amino acid residues at both the N- and C-terminal tails of TGBp2, which are exposed to the outer surface of the ER-derived granule vesicles, with a non-specific RNA-binding conformation.

The non-specific RNA binding of TGBp2 also raises the question of "how the non-specific RNA binding of TGBp2 leads to specific transport of viral RNA". It is unlikely that the functional specificity of TGBp2 is conferred by the protein components of viral RNP since TGBp1 and CP do not influence the RNA-binding property of TGBp2 (data not shown). More likely, some accessory proteins, such as TGBp3 [16] and/or certain unknown host factors associated with TGBp2 in the granular vesicles, play the role. The finding that the functional specificity of non-specific RNA-binding proteins can be achieved by assistance from the components of a regulatory complex may support this idea [25].

## Competing interests

The authors declare that they have no competing interests.

## Authors' contributions

All authors participated in planning the project. HTH performed the binding experiments. YHT and YLC provided the TGBp2 constructs. SHS, YHH and BYC participated in writing the manuscript. BYC was the leader of the project.
